# LightCUD: a program for diagnosing IBD based on human gut microbiome data

**DOI:** 10.1186/s13040-021-00241-2

**Published:** 2021-01-19

**Authors:** Congmin Xu, Man Zhou, Zhongjie Xie, Mo Li, Xi Zhu, Huaiqiu Zhu

**Affiliations:** 1 State Key Laboratory for Turbulence and Complex Systems, Department of Biomedical Engineering, College of Engineering, Peking University, Beijing, 100871 China; 2 Center for Quantitative Biology, Peking University, Beijing, 100871 China; 3 Department of Critical Care Medicine, Peking University Third Hospital, Beijing, 100191 China

**Keywords:** Machine learning algorithm, Human gut microbiome, IBD, Biomarker

## Abstract

**Background:**

The diagnosis of inflammatory bowel disease (IBD) and discrimination between the types of IBD are clinically important. IBD is associated with marked changes in the intestinal microbiota. Advances in next-generation sequencing (NGS) technology and the improved hospital bioinformatics analysis ability motivated us to develop a diagnostic method based on the gut microbiome.

**Results:**

Using a set of whole-genome sequencing (WGS) data from 349 human gut microbiota samples with two types of IBD and healthy controls, we assembled and aligned WGS short reads to obtain feature profiles of strains and genera. The genus and strain profiles were used for the 16S-based and WGS-based diagnostic modules construction respectively. We designed a novel feature selection procedure to select those case-specific features. With these features, we built discrimination models using different machine learning algorithms. The machine learning algorithm LightGBM outperformed other algorithms in this study and thus was chosen as the core algorithm. Specially, we identified two small sets of biomarkers (strains) separately for the WGS-based health vs IBD module and ulcerative colitis vs Crohn’s disease module, which contributed to the optimization of model performance during pre-training.

We released LightCUD as an IBD diagnostic program built with LightGBM. The high performance has been validated through five-fold cross-validation and using an independent test data set. LightCUD was implemented in Python and packaged free for installation with customized databases. With WGS data or 16S rRNA sequencing data of gut microbiome samples as the input, LightCUD can discriminate IBD from healthy controls with high accuracy and further identify the specific type of IBD. The executable program LightCUD was released in open source with instructions at the webpage http://cqb.pku.edu.cn/ZhuLab/LightCUD/. The identified strain biomarkers could be used to study the critical factors for disease development and recommend treatments regarding changes in the gut microbial community.

**Conclusions:**

As the first released human gut microbiome-based IBD diagnostic tool, LightCUD demonstrates a high-performance for both WGS and 16S sequencing data. The strains that either identify healthy controls from IBD patients or distinguish the specific type of IBD are expected to be clinically important to serve as biomarkers.

**Supplementary Information:**

The online version contains supplementary material available at 10.1186/s13040-021-00241-2.

## Background

Inflammatory bowel disease (IBD) is a group of inflammatory conditions of the colon and small intestine that affects over 2.5 million Europeans and 3.1 million Americans [1, 2], and has a notably increasing prevalence in the Asia-Pacific region [3]. An early accurate diagnosis can help clinicians to improve treatment. However, there is no “gold standard” diagnosis for monitoring quiescent disease in patients with IBD. Moreover, the two major types of IBD, ulcerative colitis (UC) and Crohn’s disease (CD) [4], have different mechanisms of tissue damage [5], necessitating different treatment strategies. It is clinically critical but usually difficult to identify the specific types of IBD because there are no biomarkers or clinical tests capable of discriminating CD from UC patients in practice [6]. Even colonoscopy may miss inflammation in some parts of the gastrointestinal tract [7].

The human gut microbiota has been viewed as a relatively “forgotten” organ, however, has been increasingly concerned with an important role in health [8]. Recently the next-generation sequencing (NGS)-based profiling studies of the intestinal microbiome have reinforced the view that the pathogenesis of IBD is closely associated with the unbalanced composition of the microbial community [9–11]. In contrast to serum biomarkers, fecal biomarkers respond more directly to the changes in the intestinal conditions. With the development of NGS technology and advances in hospital bioinformatics analysis, it is time to propose a diagnostic procedure to discriminate UC and CD from non-IBD colitis, especially based on the current high-throughput NGS data of the human gut microbiome.

In this work, we present a tool, named LightCUD, for discriminating UC and CD from non-IBD colitis using the human gut microbiome. LightCUD embodies four high-performance modules, namely, WGS-based health vs IBD module, WGS-based UC vs CD module, 16S-based health vs IBD module, and 16S-based UC vs CD module. Each module is composed of a machine learning model and a customized reference database. In detail, we used the high-throughput whole-genome sequencing (WGS) data to analyze the microbial composition of gut microbiota samples. These samples were from patients with UC and CD, and healthy controls. The taxonomic profiles of these samples were obtained as feature abundance matrices (FAMs) at the strain level for two WGS-based modules and genus levels for two 16S-based modules respectively. We designed a feature selection strategy for all the modules. Also, we compared the performances of five different machine learning algorithms, i.e., logistic regression, random forest, gradient boosting classifier, support vector machine, and LightGBM for training each model of the corresponding module [12–15]. The LightGBM-based models performed best. As a result, we established four high-performance lightGBM-based modules, namely, WGS-based health vs IBD module, WGS-based UC vs CD module, 16S-based health vs IBS module, and 16S-based UC vs CD module. For the two WGS-based modules, we further optimized the feature/strain sets to improve the performance of the module. The result illustrated that 49 strains for WGS-based health vs IBD module and 12 strains for WGS-based UC vs CD module could achieve the best performances. Finally, we constructed and released the tool LightCUD. With 16S rRNA sequencing or WGS data from individual gut microbiota samples as input data, LightCUD predicts the probability of having IBD, and the sample identified as IBD will then be classified as UC or CD.

## Results

### Implementation and performance of LightCUD

**Fig. 1:**
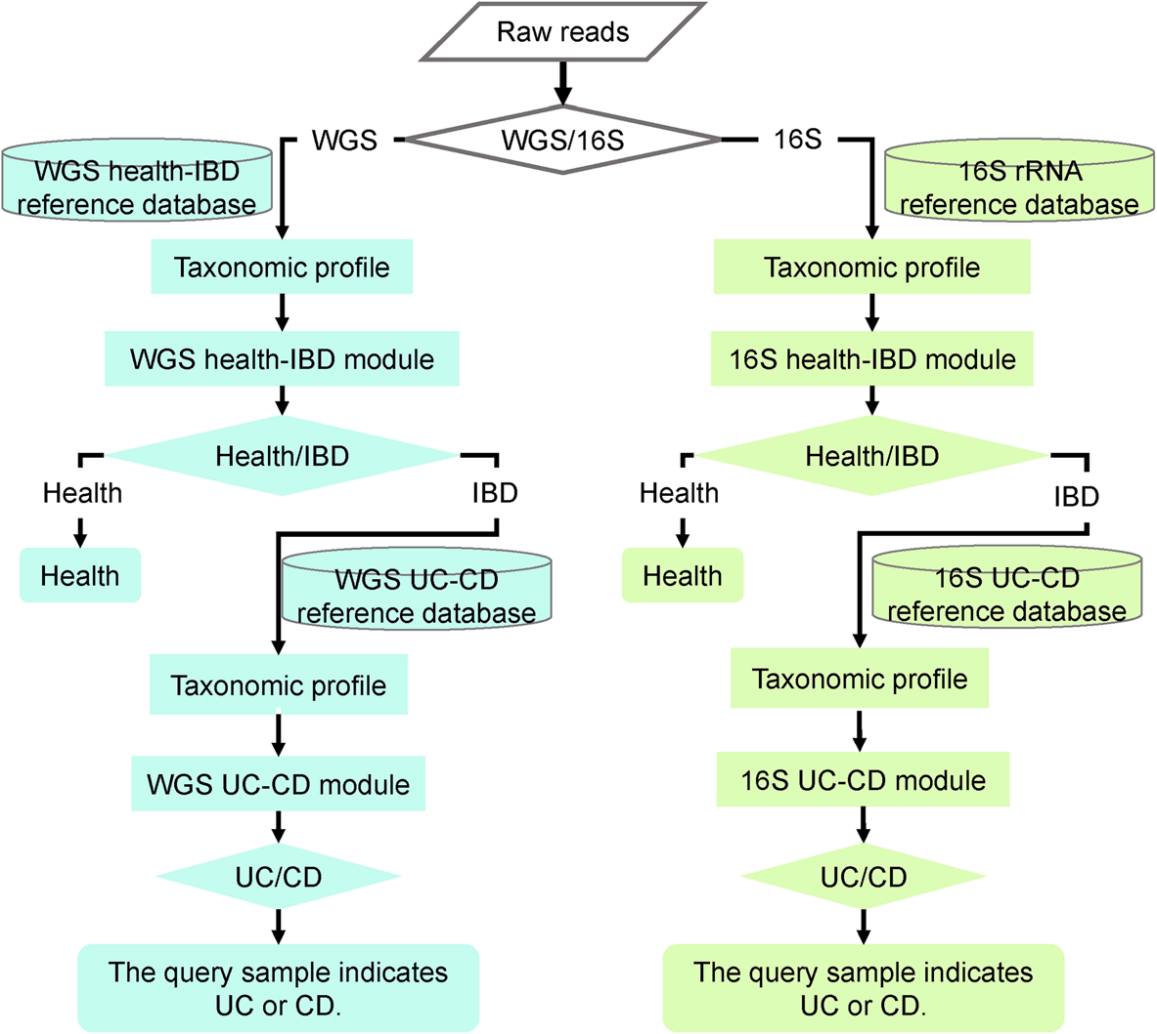
Schematic of the LightCUD framework. The input data to LightCUD is the raw reads of the sample in FASTA format. First, with the ‘-t’ parameter, LightCUD decides the data type. For different data types, different customized reference databases are used. For both WGS and 16S data, LightCUD goes through a two-stage judgment. At the first stage, LightCUD decides whether the query sample is healthy or IBD. If IBD, LightCUD further judges the specific type, namely, UC or CD

As the feature sets (genera for 16S-based modules and strains for WGS-based modules) and model parameters were determined, we trained the models of LightCUD with all the training samples to build a universal decision tree. Finally, we released the LightCUD program for first identifying IBD colitis samples and further discriminating UC and CD, for both WGS data and 16S sequencing data from the human gut microbiota samples. As shown in Fig. 1, LightCUD goes through different processing routes for WGS data and 16S sequencing data. For WGS data, LightCUD first blasts the raw data in FASTA format against the customized health vs IBD reference database embodying the reference genomes of the 49 strains we determined above. With the alignment results, LightCUD calculates the taxonomic profiles and then determines whether the query sample tends to be healthy or IBD. If IBD is indicated, LightCUD further decides whether the query sample belongs to UC or CD type in this sample using the customized WGS reference database of the 12 selected strains. For 16S data, similar to the WGS module, there are two 16S rRNA reference databases, one including 252 genera for health vs IBD task and the other one containing 91 genera for UC vs CD task. With 16S rRNA data as input, LightCUD aligns the query sequences against one of these two reference databases based on the stage of the decision to be made. Firstly, LightCUD decides whether this sample indicates health or IBD. If IBD, LightCUD further decides the specific type of IBD as UC or CD. These four modules have been integrated into an accessible pipeline and released as an open-source tool on the webpage (see http://cqb.pku.edu.cn/ZhuLab/LightCUD/), along with the customized databases. The databases were built using the reference sequences of strains from NCBI [16] for WGS-based modules and genera from RDP [17] for 16S-based modules.

**Fig. 2:**
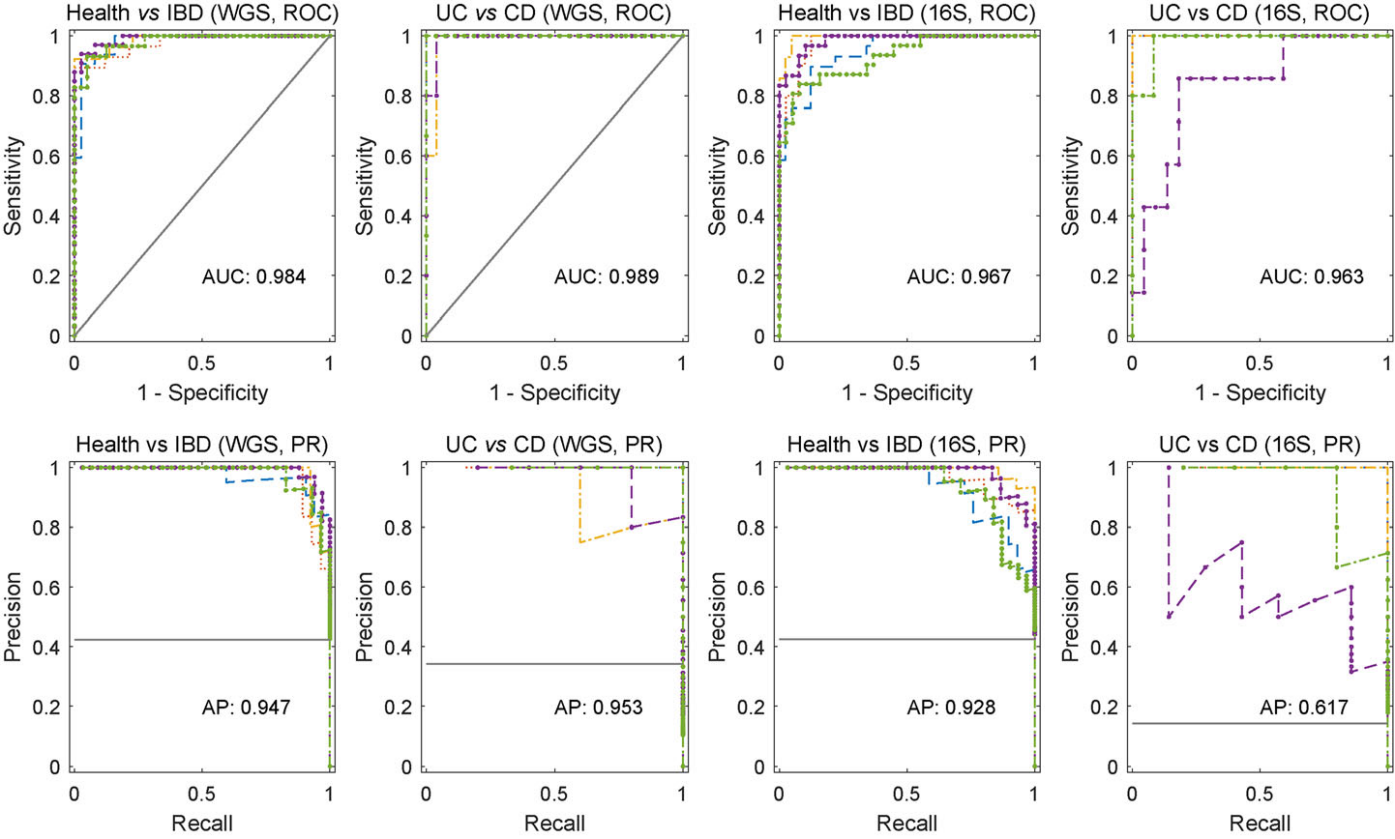
Evaluation of the performance of LightCUD. We evaluated the accuracy of disease classification using LightCUD with receiver operating characteristic curve and precision-recall curves representing the results. Lines in each subplot with different colors represent the model performance in one of the five-fold cross validations. As the training sets were unbalanced, we reported both the AUC values and the AP values. The average AUC and AP were labeled under corresponding curves

The performance of LightCUD was validated using the average area under the receiver operating characteristic curve (AUC) and average precisions (AP) with five-fold cross-validation. The average AUC and AP of both the WGS-based and the 16S-based modules, for health vs IBD and UC vs CD cases, indicated that all four cases were highly discriminative in distinguishing IBD from healthy controls, and further identifying the specific type of IBD (Fig. 2). It is also noted that the WGS-based modules (AUC = 0.984 and AP = 0.947 for health vs IBD module, AUC = 0.989, and AP = 0.953 for UC vs CD module) performed better than the 16S-based modules (AUC = 0.968and AP = 0.963 for health vs IBD module, AUC = 0.966 and AP = 0.917 for UC vs CD module). For the current release, we set default discrimination thresholds regarding the sample proportion of training data. For health vs IBD cases, default thresholds were set as *N*_IBD_/(*N*_IBD_ + *N*_Health_) = 0.42, wherein *N* represents the number of samples in the corresponding class labeled with the subscripts. Similarly, default thresholds were set as *N*_CD_/(*N*_CD_ + *N*_UC_) = 0.14 for UC vs CD cases. With the default thresholds, LightCUD reached high prediction accuracies during five-fold cross-validation, on the average, 92.3% for WGS-based health vs IBD module, 93.3% for WGS-based UC vs CD module, 88.5% for 16S-based health vs IBD module, and 93.1% for 16S-based UC vs CD module. Herein, the accuracies were the proportion of predicted labels that were the same as the actual labels of samples.

To verify the generalization ability, we further conducted blind validation with an independent dataset with 185 samples including 16 healthy controls and 169 with CD [18]. After removing low-quality reads and human genome reads, we run LightCUD on the sequences of each sample. The program returned a score indicating IBD probability (details about LightCUD aligning results in Supplementary Table 10 and Supplementary Table 11). The results showed that LightCUD maintained good performance (AUC = 0.809, AP = 0.971) in discriminating healthy controls from IBD patients with CD. Further, LightCUD showed 76.9% accuracy when discriminating CD from UC. Since samples in this test data set are geographically distinct from the training samples, this level of performance is a strong support for the robustness of LightCUD.

### The strains for WGS modules serving as biomarkers

The high performance of the strain-level WGS-based module convinced us that the 49 strains discriminating healthy controls from IBD patients and the 12 strains distinguishing the specific type of IBD were clinically valuable. More details about these strains as biomarkers are presented as follows.

**Fig. 3:**
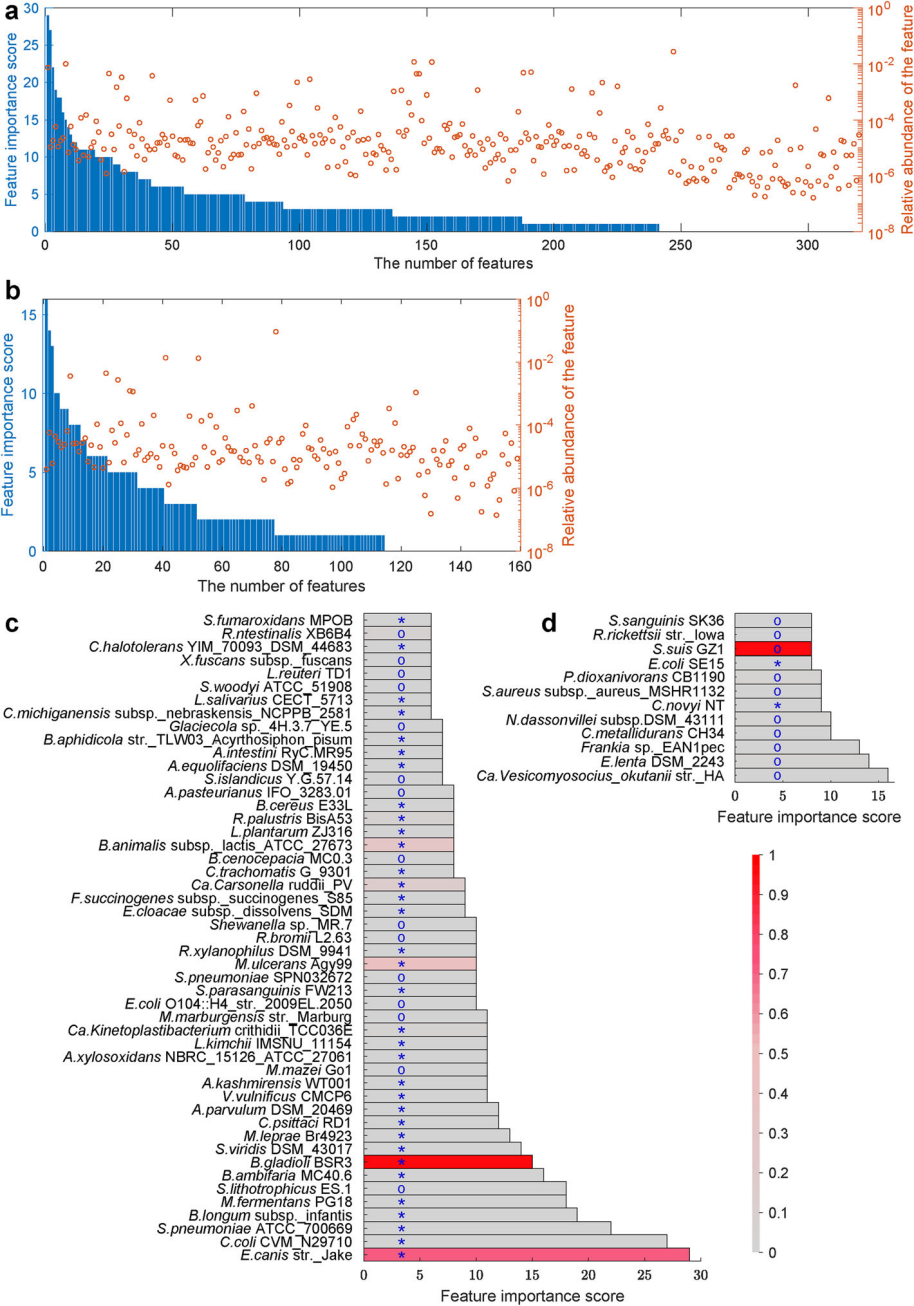
Features abundances in light of feature significance scores. Relative abundances of features for the WGS-based health vs IBD module (**a**) and the UC vs CD module (**b**). All the features that passed the three-step feature selection were shown in descending order according to feature significance score. The height of ‘o’ represents the relative abundance of corresponding strain. (**c**, **d**) Color bars show relative abundances of features, scaled to 0–1 with the maximum value of all 30 (or 15) abundances values. In (**c**), ‘*’ indicates significantly higher abundances in IBD and ‘o’ indicates the abundances of strains significantly decreased in IBD (*P* < 0.01). In (**d**), ‘*’ indicates significantly higher abundances in CD and ‘o’ indicates significantly higher abundances in UC (*P* < 0.01)

The 49 strains selected for health vs IBD discrimination and the 12 strains for UC vs CD discrimination were completely different, as shown in Fig. 3c and d. It should be noted that most of the selected disease sensitive strains were not dominant members in the microbial community. In Fig. 3a and b, the features passing the three-step selection process were sorted in descending order according to feature significance scores assigned during pre-training. The relative abundance of each feature was randomly distributed. This observation excluded the possibility that the features we finally selected were in extremely low abundances, also indicated that the features with valuable discrimination ability are not necessarily dominant.

These strains serving as discriminative biomarkers would be valuable for the analysis of IBD related intestinal microbial dysbiosis. We discussed some reported findings associated with these biomarkers. *Enterobacter cloacae*, a clinically significant species, has been reported to be enriched in the intestines of IBD patients [19]. As a member of this species, in our study strain *E. cloacae* subsp. *dissolvens* SDM was significantly enriched in IBD samples and very important for the health vs IBD discrimination (Fig. 3c). Besides, the species *Mycobacterium ulcerans* has been reported to be the major cause of the skin disease Buruli ulcer [20], herein the *M. ulcerans* strain Agy99 was enriched in IBD fecal samples and exhibited quite high discrimination ability. Furthermore, species *Burkholderia gladioli* and *Ehrlichia canis* have both been frequently reported as pathogens [21, 22], in this study the *B. gladioli* str. BSR3 and *E. canis* str. Jake with high discrimination ability was enriched in IBD samples. *Nocardiopsis dassonvillei* subsp. *dassonvillei* DSM 43111 has been reported to produce cellulases when provided with appropriate substrates, and the genome also has sequences for six predicted cellulose-degrading enzymes, which are necessary for digesting the fiber and cannot be produced within the body [23]. Cellulose has been proved effective for colitis amelioration [24]. Our results revealed that this strain was quite important for UC and CD discrimination, and was significantly depleted in CD samples compared with UC samples.

## Discussion

It should be pointed out that the WGS-based module construction involved a well-designed selection of features, which contributed to a set of highly representative features (strains) of the microbial community and accelerated the computation. The analysis of these selected features revealed the fact that highly discriminative features were not necessarily to be dominant ones. Additionally, phylogenetic tree structure indicated that these marker strains spanning a wide range of branches. Besides, these features/strains have been frequently reported as IBD associated strains. These specific strains are valuable biomarkers in designing animal models of IBD for human clinical trials to study the mechanisms of probiotics and pathogens in ameliorating inflammation.

LightCUD may be subject to the available sample sets. For example, the model distinguishing UC from CD performed inferiorly than the model distinguishing IBD from healthy controls, because of the limited number of CD samples in our training set. Even though LightCUD outperformed the other reported programs, further validation is essential before it can be used in clinical diagnosis.

## Conclusions

In this study, we constructed a diagnostic tool, LightCUD, which can discriminate IBD from healthy controls and further distinguish the specific type of IBD. The LightCUD program performed well for both WGS and 16S sequencing data, AUC > 0.95, and AP > 0.89 for all four cases (WGS-based health vs IBD and UC vs CD, 16S-based health vs IBD and UC vs CD) during five-fold cross-validation. As the first released human gut microbiome-based IBD diagnostic tool, LightCUD embodies discrimination modules constructed with stool samples better than any other reported classifiers. Gevers et al. constructed a classifier to distinguish CD from healthy controls based on 199 stool samples with an AUC of 0.66 [9]. Papa et al. performed an analysis of the 16S sequencing data from 91 stool samples and reached a discrimination accuracy with an AUC of 0.83 [17]. For the current study, LightCUD has only a single command line but provides a non-invasive mechanism of distinguishing IBD from healthy controls based on stool samples. For either WGS or 16S data, LightCUD processes a sample in several hours, depending on the sequencing depth. The parallel computation may further reduce the running time of prediction. With the development and popularity of NGS, LightCUD highlights the potential of diagnostic tools developed with machine learning algorithms based on the data of human gut microbiome.

## Methods

**Fig. 4:**
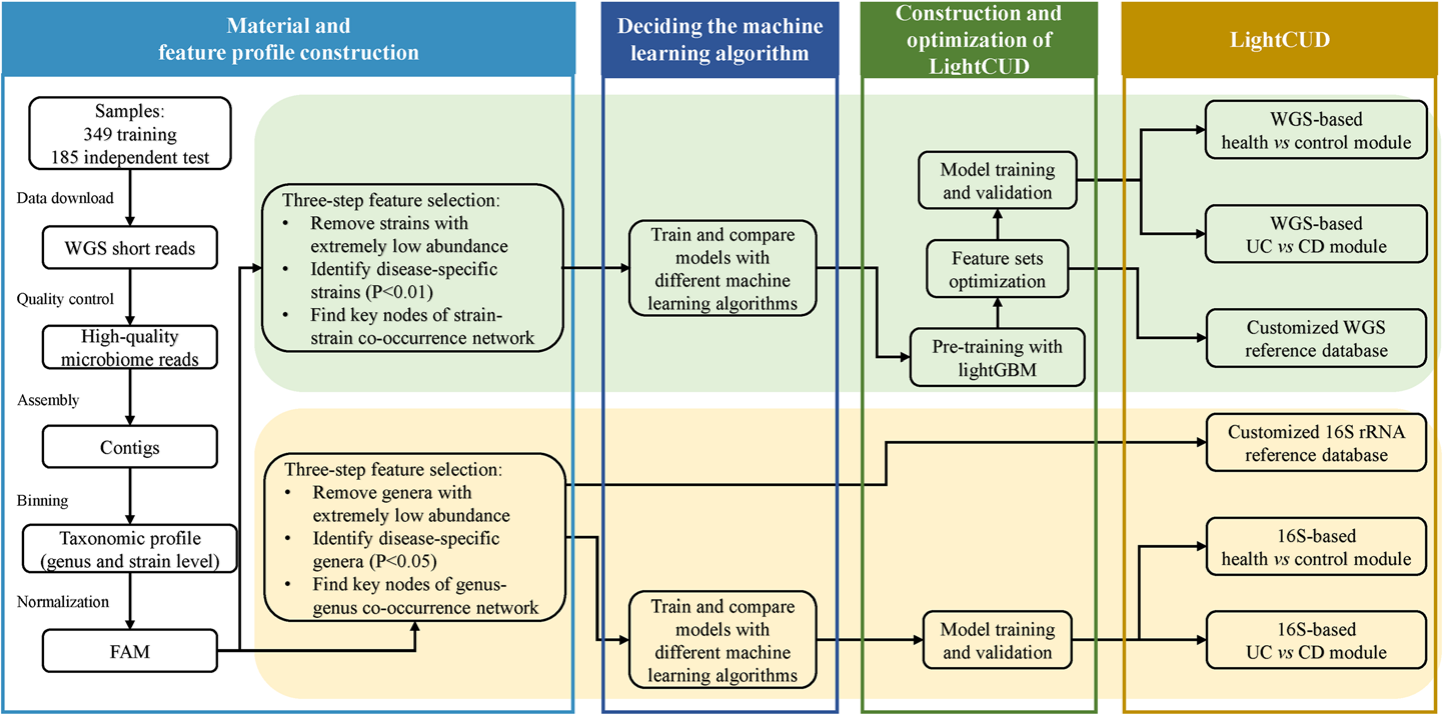
The pipeline of data processing and the LightCUD program construction. With WGS raw data of 349 samples, we eliminated the low-quality reads and assembled the remaining reads into contigs. Contigs > 1000 bp were taxonomically binned into strains and genera. 16S rRNA-based discrimination modules were constructed with genus-level profiles and WGS-based discrimination modules were constructed with strain-level profiles. For the four modules, we designed different feature selection procedures and compared different machine learning algorithms. LightGBM was selected as the core algorithm for modules construction for its best performance. For WGS-based modules, we further optimized the model by shrinking the feature set through pre-training. Finally, a high-performance dual-usage discrimination program LightCUD was successfully constructed. The corresponding reference databases were released along with the prediction modules

As shown in Fig. 4, we first conducted a metagenomics analysis for WGS data of human gut microbiota samples from two types of IBD and healthy controls. Based on the alignment result from the last step, we constructed feature profiles at the strain level for WGS-based modules, and at the genus level for 16S-based modules. After the well-designed feature selection steps, we selected the LightGBM models to construct the discriminant modules, outperformed the other four machine learning algorithms. We then describe in detail the methods.

### Sample description

We downloaded a deeply sequenced microbiota data set of 396 human stool samples from a public database, which has been described in a previous study of the human intestinal tract metagenome [25]. There are 396 samples from 318 individuals (Supplementary Table 1). There are 78 individuals sequenced twice. Among the samples, 47 ones labeled with relative health were excluded because of their uncertainty of being IBD patients or healthy (see details in Supplementary Table 1). After removing those relative healthy individuals, we have 271 individuals and 349 samples left. To remove the potential inflation induced by those samples from individuals sequenced twice, we split individuals instead of samples during k-fold cross validation. The samples from the same individual were together treated as either training samples or test samples. A total of 349 samples were included in this study, consisting of 201 samples from healthy controls, 127 samples from UC, and 21 samples from the CD. 4.68 TB WGS paired-end short reads of these samples were downloaded from NCBI GenBank [16]. We first assembled the original short reads into contigs using InteMAP [26], which was designed as an integrated assembly pipeline for NGS metagenomic short reads. To ensure the validity of the further analysis, short reads with low quality and contigs shorter than 1000 bp were filtered out. The evaluation result of the assembled contigs is described in Supplementary Table 2.

To verify the generalization ability of LightCUD, we also conducted blind validation with an independent data set, which has been described in another study [18]. This data set includes 244 GB short reads of 185 samples with a moderate size, including 16 healthy controls and 169 with CD (> 696 MB and < 2000 MB per sample, filtering out samples in the bottom or top quartiles were filtered out, details of the samples in Supplementary Table 3).

### Constructing feature profiles for WGS-based and 16S-based modules

NGS techniques enable us to systematically characterize the composition of a complex microbial community, such as human gut microbiota. 16S rRNA genes for bacteria were the most commonly used target genes for molecular analysis, which provides fairly consistent taxonomic assignment for a relatively wide range of genera [27]. Although expensive, WGS can theoretically classify taxonomic composition at the strain level. WGS-based strain typing is widely used in the epidemiologic analysis of bacterial pathogens in public health, so we developed our program with both WGS-based and 16S rRNA-based modules. In this subsection, we then describe how to construct the strain profiles and genus profiles as FAMs for the WGS-based modules and 16S-based modules separately.

With high-quality contigs, we were then able to recognize the members of the microbial community. To perform taxonomic binning at the strain level, we used the 2712 strain genomes references in NCBI RefSeq [16]. The PhymmBL tool [28] was applied to taxonomic binning, which combined the sequence composition-based method and sequence alignment algorithm. With Bowtie 2–2.1.0 alignment [29], phylogenetic profiles for a sample were then calculated by counting the number of short reads aligned to each contig. To obtain comparable relative abundances of strains, a correction process for sequencing depth was applied during which the numbers of aligned reads were normalized by the contig length and the number of matches per sample. The resulting values of relative abundances were between 0 and 1 (alignment details in Supplementary Table 4; annotated strains in Supplementary Table 5). The strain-level taxonomic profiles served as FAMs for WGS-based modules.

In the current study, we also consider such a case that only 16S rRNA data are sequenced for human gut microbiota samples. For this case, we designed 16S-based modules trained with the WGS data herein and calculated the genus-level taxonomic profiles as FAMs. The relative abundance of each genus in the 16S-based FAM was calculated by adding up the relative abundances of strains belonging to this genus. The annotated genera were listed in Supplementary Table 6.

For WGS-based modules, we finally annotated 2661 strains as features with the data set of 349 samples (Supplementary Table 5). As we know, a major drawback of WGS analysis is that it is very expensive, mainly because of the large size of the whole-genome reference database. To address this issue, we optimized the feature set by selecting the most discriminative, so that we could construct a relatively small reference database meanwhile avoiding model overfitting. The features selection process consisted of three steps as follow:

(i) Only strains with relative abundances more than 10^− 6^ in at least one sample was reserved. This step was designed to filter out some features that might be noise information for model training.

(ii) Group versus group comparative analysis was carried out and strains that significantly passed the Wilcoxon rank-sum test (*P* < 0.01) were retained [30]. This analysis is commonly used in metagenomics analysis to identify potentially disease-related taxa. We added this step to select case sensitive strains, and avoid the noise created by insensitive features.

(iii) The hub nodes/strains in the strain-strain co-occurrence network graphs were selected in an iterative procedure. In co-occurrence networks, nodes were strains, and links represented validated strain-strain correlations (*P* < 0.05). The correlations were calculated using the Spearman correlation in SparCC [31], based on the relative abundances of strains across samples. The most intensively connected strain was picked out as the representative strain (or feature) in each iteration. The selected strain and its strong connected strains (|*R*| > 0.4) were then removed from the graph and the remaining strains were iterated into the next loop of feature selection. This process was kept running until less than three nodes were left in the network graph. This step was designed to select the most representative strains and exclude the strains intensively correlated with the representative strains.

We conducted this feature selection process separately for the WGS-based both healthy vs IBD and UC vs CD cases. Finally, we have 320 strains for health vs IBD case, and 159 strains for UC vs CD case, of which 29 overlapped strains were good discriminators for both cases. We listed these strains/features in Supplementary Table 7.

For the 16S-based module, 508 genera were annotated as features (Supplementary Table 6). To avoid overfitting, we conducted three-step feature selection process for 16S-based modules as well. Therefore, we have 252 features left for the 16S-based health vs IBD module and 91 features left for UC vs CD modules.

### Deciding the machine learning algorithm for building LightCUD

The feature selection step mentioned above were applied on training samples only during each round of five-fold cross-validation. With those selected features (strains for WGS-based modules and genera for 16S-based modules), we were able to train the machine learning models of WGS-based modules and 16S-based modules as discrimination methods with five common-used machine learning algorithms and evaluate their performances. The five algorithms are logistic regression, random forest, gradient boosting classifier, support vector machine, and LightGBM [12–14]. Herein the performances were evaluated with five-fold cross-validation using the average AUC and AP. AP was adopted as a supplementary measure since the training datasets were unbalanced. For the health vs IBD case, we have 349 samples consisting of 148 IBD and 201 healthy controls. For the UC vs CD case, we have 148 samples containing 127 UC and 21 CD. As shown in Table 1, the models built with LightGBM performed overall better than the other four algorithms, with the highest AUC in all the discrimination tasks and the highest AP in three out of four discrimination tasks (WGS-based health vs IBD and UC vs CD, 16S-based health vs IBD and UC vs CD).

**Table 1:** Comparison of model performances built with five different machine learning algorithms. LightGBM performed better than the other four algorithms, with the highest AUC in all the four discrimination tasks (health vs IBD and UC vs CD) and the highest AP in three out of four tasks

Discrimination tasks	WGS Health vs IBD		WGS UC vs CD		16S Health vs IBD		16S UC vs CD	
Machine learning algorithm	AUC	AP	AUC	AP	AUC	AP	AUC	AP
**Logistic regression**	0.791	0.693	0.741	0.317	0.650	0.579	0.780	0.328
**Random forest**	0.941	0.887	0.935	0.486	0.909	0.855	0.931	0.498
**Gradient boosting classifier**	0.784	0.713	0.797	0.271	0.739	0.650	0.830	0.359
**SVM**	0.837	0.733	0.782	0.329	0.775	0.708	0.863	0.289
**LightGBM**	**0.964**	**0.955**	**0.942**	**0.848**	**0.968**	**0.963**	**0.966**	**0.917**

### Construction and optimization of the LightCUD method

After determining LightGBM as the core machine learning algorithm, we then present the construction details of LightCUD. For the two 16S rRNA modules, we trained the model parameters with the taxonomic profiles of 252 (health vs IBD) and 91 (UC vs CD) genera passing the feature selection. With the genus profiles of 201 healthy controls and 148 IBD patients, we trained 16S-based health vs IBD module. With the genus profiles of 127 UC and 21 CD, we trained the 16S-based UC vs CD module. The model parameters were tuned to optimize the performance of the two cases through five-fold cross-validation. The relatively short reference sequences of the 16S rRNA reference database allowed for the rapid alignment of query sequences, so the 16S modules could work very fast and make a judgment for one sample in a few minutes.

For WGS data, we constructed and optimized the WGS-based modules with strains as features. Compared with genera, strains are remarkably more critical for medical interest in judgment of pathogenicity and characterization of disease. The three-step feature selection strategy reduced the number of features/strains. However, the database including reference genomes of hundreds of strains was still too large for application, so we further shrunk the feature set using a pre-training procedure. At the same time, this procedure could improve the performance and stability of the WGS-based modules.

**Fig. 5:**
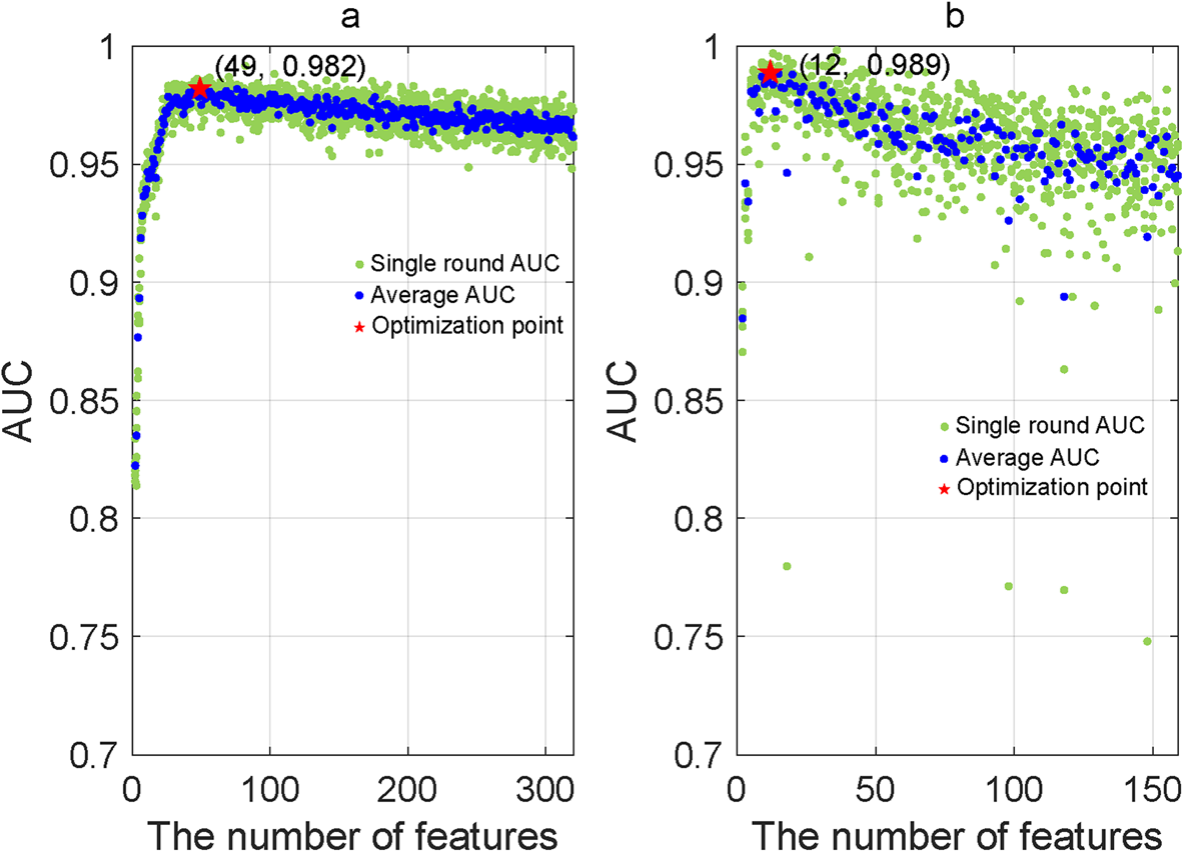
Optimizing the feature sets for WGS-based modules. The cyan dots denoted the AUC values for a different round of five-fold cross validation with the different number of features, and the blue dots represented mean values of the cyan dots in the same column. (**a**) Illustrated the WGS-based health vs IBD case and (**b**) illustrated the WGS-based UC vs CD case. For both the cases, AUCs increased with more features at the beginning and decreased after reaching the top values. Forty-nine features for WGS-based health vs IBD case and 12 features for UC vs CD case were best

Through pre-training, we assigned significance scores to the features/strains that passed the three-step feature selection procedure. The significance score of each strain was calculated using LightGBM through evaluating the increment of the error rate caused by removing that strain from the set of predictors. To optimize the generalization ability, the size of the feature set was determined, and the most important features were selected as follows. All the features/strains were sorted in a queue according to their significance scores. Herein we added the top one feature into a waiting list and evaluated the AUC of the models using the feature in the waiting list with five-fold cross-validation withing the same set of training samples used for feature selection. Then, we added the next feature to the waiting list and calculated the AUC again. This process was continued until all the features were added to the waiting list. For the final release version, there are overall 320 strains for health vs IBD case, and 159 strains for UC vs CD case. We found that with an increasing number of features added to the waiting list, the AUCs increased at the beginning and decreased after reaching the largest values. The WGS-based health vs IBD module with 49 features achieved the best performance, and the UC vs CD module with 12 features achieved the best performance (Fig. 5). The module AUCs labeled as stars in Fig. 5 were higher than those of modules shown in Table 1, even though the number of feature/strains are reduced, which illustrated that the model performances was improved by optimizing the feature set, owing to the reduction of potential noise features. Therefore, for WGS-based modules, we selected the 49 most important features/strains for health vs IBD discrimination and the 12 strains for UC vs CD discrimination. Relative abundances of these strains across corresponding samples were listed in Supplementary Tables 8 and 9. With these features, we separately trained the health vs IBD module and UC vs CD module.

## Supplementary Information

**Additional file 1: Supplementary Table 1.** Sample information. **Supplementary Table 2.** Assembly result evaluation of the training short reads. **Supplementary Table 3.** Test samples. **Supplementary Table 4.** Ratio of reads mapped to annotated contigs. **Supplementary Table 5.** Annotated strains from the training data. **Supplementary Table 6.** Annotated genera from the training data. **Supplementary Table 7.** Features/strains left after three-step feature selection. **Supplementary Table 8.** Forty-nine most important features/strains for the WGS-based module for health vs IBD. **Supplementary Table 9.** Twelve most important features/strains for the WGS-based module for UC vs CD. **Supplementary Table 10.** Taxonomic profile of test samples with LightCUD WGS-based health vs IBD module. **Supplementary Table 11.** Taxonomic profile of test samples with LightCUD WGS-based UC vs CD module.

## Data Availability

The data we used in this paper was downloaded from a previously published paper [18, 25]. The data we generated during data analysis was released as additional files.

## Abbreviations

IBD: Inflammatory bowel disease
WGS: Whole-genome sequencing
UC: Ulcerative colitis
CD: Crohn’s disease
NGS: Next-generation sequencing
FAM: Feature abundance matrices
AUC: Area under the receiver operating characteristic curve
AP: Average precisions
